# The Microfluidic Probe: Operation and Use for Localized Surface Processing

**DOI:** 10.3791/1418

**Published:** 2009-06-04

**Authors:** Cecile M. Perrault, Mohammad A. Qasaimeh, David Juncker

**Affiliations:** Department of Biomedical Engineering, McGill University

## Abstract

Microfluidic devices allow assays to be performed using minute amounts of sample and have recently been used to control the microenvironment of cells. Microfluidics is commonly associated with closed microchannels which limit their use to samples that can be introduced, and cultured in the case of cells, within a confined volume. On the other hand, micropipetting system have been used to locally perfuse cells and surfaces, notably using push-pull setups where one pipette acts as source and the other one as sink, but the confinement of the flow is difficult in three dimensions. Furthermore, pipettes are fragile and difficult to position and hence are used in static configuration only.

The microfluidic probe (MFP) circumvents the constraints imposed by the construction of closed microfluidic channels and instead of enclosing the sample into the microfluidic system, the microfluidic flow can be directly delivered onto the sample, and scanned across the sample, using the MFP. . The injection and aspiration openings are located within a few tens of micrometers of one another so that a microjet injected into the gap is confined by the hydrodynamic forces of the surrounding liquid and entirely aspirated back into the other opening. The microjet can be flushed across the substrate surface and provides a precise tool for localized deposition/delivery of reagents which can be used over large areas by scanning the probe across the surface. In this video we present the microfluidic probe^1^ (MFP). We explain in detail how to assemble the MFP, mount it atop an inverted microscope, and align it relative to the substrate surface, and finally show how to use it to process a substrate surface immersed in a buffer.

**Figure:**
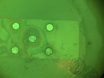


## Protocol

### 1. Microfabrication of the probe head ( process not shown in the video)

A Si^2^wafer , four inches in diameter, 525 μm thick, with a 1 μm thick thermal SiO2 layer is spincoated with a photoresist (PR) for 45 s at 4000 rpm.
The wafer is prebaked at 110C for 50 s, and exposed through a mask featuring all the elements (ports and microchannels) for 5 s, developed and rinsed in DI.
The uncovered SiO2 is etched away in a 1:7 buffered hydrofluoric acid (BHF) solution in ≈15 min (dewetting of the substrate where SiO2 has been etched indicates completion of the etch). An O2 plasma or acetone is used to ash or strip the remaining PR.
A second PR layer is spin-coated at 1500 rpm for 45 s, yielding a thick overlayer of ≈10 μm [31]. The SiO2 pattern underneath this PR layer is still visible and is used to align the wafer with a second mask featuring the ports only.
After exposure and development of the PR, the wafer is rinsed, dried, and postbaked at 95C for 20 min.
The Si wafer is attached onto a support wafer with melted white wax to protect the chuck.
An inductively coupled plasma (ICP) DRIE is used to transfer the PR and embedded SiO2 patterns into wafer topography in a three-step process:
  DRIE to make≈500μmdeep ports into Si (pattern defined by the thick PR).
  Without unloading the wafer from the DRIE machine, the PR is ashed using a plasma.
  The exposed SiO2 pattern acts as mask for a second dry etch process, creating 50 μm deep channels, and opening the filling and venting ports through the wafer. After unloading, the support wafer is detached under a stream of warm water. The micromachined wafer is then cleaned with acetone, ethanol and DI.
Individual MFP chips are diced.
A PDMS interface block is fabricated by casting into a micromould composed of two structured poly(methylmethacrylate) (PMMA) elements, a polished steel plate forming the bottom, and two capillaries (each inserted into one of the two vias—access holes—in the steel plate) serving as place holders for the fluidic connection holes. The PDMS is cured in an oven at 60C for at least 1 h.
The PDMS block is bonded to a diced MFP silicon chip by activating both parts in air plasma at 1 mbar for 24 s at 230W, and joining the two together using a home-made mechanical alignment aid.
The assembly is left to bond in a 60C oven for a minimum of 1 hour

### 2. Assembly of the MFP

Gas-tight glass syringes are filled with the appropriate reagents using plastic syringes and needles to ensure that no air bubbles are present. Typically, we use a 1 -10 microliter syringe for injection, and a syringe with 5-10 times larger volume for aspiration.
The syringes are connected to capillary tubing using Nanotight fittings with low dead volume.
Capillaries are filled and checked for bubbles under the microscope.
The MFP chip is prefilled with buffer solution to prevent trapping of bubble when connecting the capillaries.
The capillaries are plugged into the PDMS connection piece in the probe head

### 3. Set-up of the MFP

The probe head is clamped into the probe holder and mounted on the probe station atop an inverted microscope
The syringes are placed into high-precision syringe pumps.
The substrate, such as a glass slide, is inserted into a home-made holder that is affixed to the microscope stage.
The parallelism of the mesa of the MFP and the substrate is adjusted using a pair of goniometers by observing the Newton’s rings (interference fringes) that appear when the MFP is brought into contact with the substrate. The point of contact and the frequency of the rings serve as indication of the tilt. When the MFP is aligned with the surface, a single interference ring extends over the entire surface. This measure also serves to calibrate the separation between MFP and substrate.
The gap between the MFP and the substrate is critical for surface patterning processes. Because the substrate is processed by scanning it below the MFP, the horizontal alignment has to be adjusted with micrometer precision and is achieved using a three point support formed by three micrometer screws.

### 4. Operation of the MFP

Dispensing is controlled via LabView software. Device operation is visualized by eye and using a CCD camera. The injection:aspiration ratio varies from 1:3 to 1:10, depending on the diffusivity of the reagent with the surrounding buffer and the desired geometrical flow pattern.
 To check for proper operation of the aspiration syringe and the presence of bubbles, first inject liquid with the aspiration syringe before starting proper aspiration.
Start injection of liquid and monitor flow and confinement of beads or of fluorescent tracer dye.
Use the probe for the particular application, i.e. scan across surface for processing for deposition, etching or staining of surface or cells.

## Discussion

The microfluidic probe (MFP) is versatile because it is (i) mobile, (ii) adaptable for use with different types of reagents and substrate and it can (iii) be operated over large areas.

Unwanted bubbles can lead to disruption of the flow To avoid bubbles, all components need to be filled with liquids prior to assembly.  The gap between the probe and the surface is only a few micrometers, yet the mesa is several hundred micrometers wide, and distances in the range of centimeters are scanned. Therefore both horizontality of the scanned surface and the parallelism between the MFP mesa and the substrate need to be adjusted with great care. Finally, the ratio between aspiration and injection has to be large enough to capture all of the reagent injected into the gap between the MFP and the substrate.

The MFP may be used for patterning surfaces with proteins under mild conditions, to process tissues or  individual cells immersed in physiological buffers, or to etch patterns into a surface.
